# Comparative bioinformatics analysis between proteomes of rabbit aneurysm model and human intracranial aneurysm with label‐free quantitative proteomics

**DOI:** 10.1111/cns.13570

**Published:** 2021-01-03

**Authors:** Yingjun Liu, Yaying Song, Peixi Liu, Sichen Li, Yuan Shi, Guo Yu, Kai Quan, Zhiyuan Fan, Peiliang Li, Qingzhu An, Wei Zhu

**Affiliations:** ^1^ Department of Neurosurgery Huashan Hospital Shanghai Medical College Fudan University Shanghai China; ^2^ Neurosurgical Institute of Fudan University Shanghai China; ^3^ Shanghai Clinical Medical Center of Neurosurgery. Shanghai China; ^4^ Shanghai Key Laboratory of Brain Function and Restoration and Neural Regeneration Shanghai China; ^5^ Department of Neurology Renji Hospital of Shanghai Jiao Tong University Shanghai China; ^6^ Neuroscience and Neuroengineering Research Center Med‐X Research Institute and School of Biomedical Engineering Shanghai Jiao Tong University Shanghai China

**Keywords:** bioinformatics, focal adhesion, intracranial aneurysm, proteomics, rabbit elastase‐induced aneurysm

## Abstract

**Aims:**

This study aimed to find critical proteins involved in the development of intracranial aneurysm by comparing proteomes of rabbit aneurysm model and human aneurysms.

**Methods:**

Five human intracranial aneurysm samples and 5 superficial temporal artery samples, and 4 rabbit aneurysm samples and 4 control samples were collected for protein mass spectrometry. Four human intracranial aneurysm samples and 4 superficial temporal artery samples, and 6 rabbit aneurysm samples and 6 control samples were used for immunochemistry.

**Results:**

Proteomic analysis revealed 180 significantly differentially expressed proteins in human intracranial aneurysms and 716 significantly differentially expressed proteins in rabbit aneurysms. Among them, 57 proteins were differentially expressed in both species, in which 24 were increased and 33 were decreased in aneurysms compared to the control groups. Proteins were involved in focal adhesion and extracellular matrix‐receptor interaction pathways. We found that COL4A2, MYLK, VCL, and TAGLN may be related to aneurysm development.

**Conclusion:**

Proteomics analysis provided fundamental insights into the pathogenesis of aneurysm. Proteins related to focal adhesion and extracellular matrix‐receptor interaction pathways play an important role in the occurrence and development of intracranial aneurysm.

## Graphical abstract

Using proteomic analysis and cell model to verify the protein expression between rabbit and human aneurysm, by comparing the protein profiles between these two, looking for common genes and analyzed with bioinformatics tools, can illustrate the critical proteins involves in the development of intracranial aneurysm.

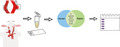

## 1 INTRODUCTION

Intracranial aneurysm (IA) is an abnormal pathological dilatation of the intracranial artery wall. Unruptured IAs occur in 3% of the adult population.^1^ Not every IA shows a tendency to rupture, and the annual incidence of IA rupture is approximately 1%.^2^ However, the mortality of aneurysmal subarachnoid hemorrhage (aSAH) remains 25%‐50%.^3^, ^4^ Intracranial endovascular treatment was invented in 1990 and has been widely used. Compared to neurosurgical treatments, the pooled clinical complication risk ^5^ and 5‐year death risk ^6^ of endovascular treatment are lower. Therefore, endovascular therapy has emerged as the preferred treatment for IA. With the popularization of interventional techniques, novel materials and treatment strategies are rapidly evolving. Rodent models of IA are mature,^7^ but are not suitable for angiography procedures. The rabbit elastase‐induced aneurysm model was first introduced in 2000,^8^ modified in 2004,^9^ and has proved to be a reliable model for research on endovascular techniques.

Previous studies have analyzed human ruptured and unruptured IA using microarrays,^10^ mRNA sequencing,^11^ and quantitative proteomics analysis.^12^ Certain pathways and genes have been identified to be highly correlated with IAs and rabbit elastase‐induced aneurysms. The rabbit elastase‐induced aneurysm model has been characterized with the above methods as well.^13^ Some studies on gene expression in the rabbit aneurysm model have been reported. However, most of them were at the transcription level, not at the protein level. Kadirvel et al. ^14^ utilized mass spectrometry to analyze protein expression in rabbit aneurysm after embolization to explore biological mechanisms of healing process in IA. However, currently, there is no suitable model that faithfully mimics the human aneurysm pathophysiology.

In this study, we aimed to compare the proteomes of rabbit aneurysms and human IAs to identify pivotal genes that are important in the pathogenesis of IA. We found several proteins whose expression levels were significantly different, such as MYLK, VCL, and collagen type IV, in both rabbit and human aneurysms, suggesting that these proteins may be vital in the development of aneurysms.

## 2 METHODS

### 2.1 Rabbit aneurysm model and sample collection

The study was reviewed and approved by the Ethics Committee of Huashan Hospital, Fudan University (2017–263). Animal experiment protocols were approved by the Department of Laboratory Animal Science at Fudan University, Shanghai, China (201802041S).

Saccular aneurysms were created in New Zealand white rabbits as described in our previous study.^15^ The right common carotid artery (CCA) was exposed, and a J‐shaped aneurysm clip was used to clamp the bifurcation of the right subclavian artery and the right CCA. Elastase was then injected to digest the right CCA. After 20 minutes of incubation, elastase was withdrawn, and the J‐shaped aneurysm clip was released. Digital subtraction angiography (DSA) was performed 30 days after aneurysm induction to evaluate the formation of aneurysms. We operated on ten rabbits, and aneurysms were successfully induced in all animals. Each experimental pair contained the aneurysm group (AN; right CCA) and the corresponding control group (CN; normal CCA). The 10 rabbits were randomly assigned into 2 groups for proteomics (4 pairs) and immunohistochemistry (IHC; 6 pairs) analyses. The right CCA (aneurysm body) was collected as the experimental group and the normal CCA as the control group.

### 2.2 Clinical tissue sample collection

Nine IA patients who underwent neurosurgical treatment were enrolled in our study, along with 9 patients who underwent neurosurgical treatment with unavoidable superficial temporal artery (STA) injury. All IA patients were diagnosed as IA for the first time without a history of rupture. Five IA and 5 STA samples were used for label‐free proteomic analysis and immediately frozen at −80°C. The rest 4 IA and 4 STA samples were fixed with 4% paraformaldehyde (Sinopharm Chemical Reagent) for IHC analysis.

### 2.3 Label‐free quantitative proteomics analysis

Four pairs of rabbit aneurysm and control samples, and 5 human IA and 5 STA samples were used for label‐free quantitative proteomics analysis. Steel balls and protein lysate were added into the samples, and a tissue grinder was used to vibrate and grind the samples at a low‐temperature environment until the tissue was completely broken. Proteins were then lysed and quantified by fluorescent peptides. According to the quantitative results, 1 μg of each digested product was analyzed by LC‐MS/MS, and each sample was analyzed once. Nanoliter flow HPLC liquid system EASY‐nLC1000 was used for separation. Enzymolysis products were separated by capillary high‐performance liquid chromatography and then analyzed by mass spectrometry using a Fusion mass spectrometer (Thermo Fisher). Eight rabbit LC‐MS/MS original files and 10 human LC‐MS/MS original files were imported into MaxQuant software separately (version 1.6.0.1) for database search using the search engine Andromeda and LFQ non‐standard quantitative analysis. The database was downloaded from the UniProt database. The anti‐base of uniprot‐Homo sapiens was used to calculate the false positive rate (FDR) of peptide and protein. MaxQuant software integrated the LFQ algorithm by extracting the isotope peaks of each peptide in each analysis, and the MaxQuant platform calculated the protein ratio using the median of the common peptide ratios in all analyses, which represented the ratio of protein ratios. The "peptides.txt" and "proteinGroups.txt" files obtained from MaxQuant were imported into Perseus software (version 1.5.1.6) for further analyses.

### 2.4 Bioinformatics analysis

Gene Ontology (GO) classification was used to elucidate the relationship among different genes. Three categories were established: biological process, molecular function, and cellular component. Proteins were further divided into sub‐categories under these three categories according to their respective characteristics. Profile and cluster analyses were performed using g:Profiler. Bioinformatic analysis was conducted through the Kyoto Encyclopedia of Genes and Genomes (KEGG) pathway analysis. Protein‐protein interactions (PPIs) were analyzed with STRING.

### 2.5 Immunohistochemistry of human and rabbit tissue samples

The samples were fixed in 4% paraformaldehyde and embedded in paraffin. Paraffin‐embedded tissues were dewaxed, followed by antigen retrieval. Sections were blocked by bovine serum albumin (BSA; Sinopharm Chemical Reagent) for 30 minutes after blocking endogenous peroxidase. Sections were then incubated with mouse anti‐vinculin (VCL, 1:200 dilution; MilliporeSigma, United States) and rabbit anti‐COL4A2 (1:200 dilution; Bioss, China) overnight. The corresponding secondary antibody (Dako, Denmark) was added to the tissues and incubated for 50 minutes at room temperature. Freshly prepared DAB coloring solution (Dako) was then added, and the color development time was determined after observation under a microscope. Excessive color was washed with tap water to terminate the color development. Finally, nuclei were counterstained, and the sections were dehydrated and sealed.

### 2.6 Rat aortic smooth muscle cell isolation

Aortic smooth muscle cells (SMCs) were obtained from male Sprague‐Dawley rats (4–6 weeks of age). The thoracic aorta was harvested, and tissue segments were washed for three times using phosphate‐buffered saline (PBS; Hyclone, United States) with 5% penicillin/streptomycin (Hyclone). The adventitia layer was carefully separated under a microscope, and the endothelial layer was gently removed with tweezers. The tissues were then cut into 1×1 mm fragment and seeded into 6‐mm dishes with Dulbecco's modified eagle medium (DMEM; Hyclone) supplemented with 20% fetal bovine serum (FBS; Gibco, United States) and 1% penicillin/streptomycin. The tissue fragments were incubated in a humidified atmosphere of 5% CO_2_ and 95% air at 37°C for 2–4 weeks. Cells were digested and seeded into 10‐cm dishes with DMEM supplemented with 10% FBS and 1% penicillin/streptomycin after growing from the tissues and reaching 70%‐80% confluence. SMCs were verified with a rabbit anti‐alpha‐smooth muscle actin (α‐SMA) antibody (Abcam).

### 2.7 Cell viability assay

Rat aortic SMCs were seeded onto 96‐well plates at a density of 5 × 10^3^ cells per well in triplicate. Cells were incubated in serum‐free medium when reaching 70%‐80% confluence. Twenty‐four hours later, the cells were treated with tumor necrosis factor‐alpha (TNF‐α; Peprotech, United States) at different concentrations (0, 0.1, 1, 10, and 40 ng/ml) for 2 hours. Cell viability was measured using a CCK‐8 assay kit (Dojindo, Japan). One hundred microliter of CCK‐8 was added into each well and incubated for 2 hours at 37°C according to the manufacturer's instructions. The absorbance was measured at 450 nm with a spectrometer.

### 2.8 Western blotting analysis

Proteins were lysed from rat aortic SMCs, and equal amount of protein per lane (20 μg) was separated by 12.5% SDS‐PAGE gel (Epizyme, China). Proteins were electrotransferred onto a polyvinylidene difluoride (PVDF) membrane (MilliporeSigma). The membrane was blocked with a QuickBlock blocking buffer (Beyotime Biotechnology) for 15 minutes at room temperature, followed by incubation with different primary antibodies, including rabbit anti‐MYLK (1:5000 dilution; Abcam), rabbit anti‐COL4A2 (1:500 dilution; Bioss, China), rabbit anti‐SMA (1:100 dilution; Abcam), goat anti‐ transgelin (TAGLN, 1:500 dilution; Abcam), and mouse anti‐VCL (1:1000 dilution; Millipore) overnight. The membrane was then incubated with horseradish peroxidase‐conjugated secondary antibody for 1 h at room temperature. Immunoblots were probed using enhanced ECL substrate (Thermo). The blot was detected using an imaging system (Bio‐Rad), and the chemiluminescence level was recorded. The results were normalized to GAPDH. The experiments were replicated for three times.

### 2.9 Statistical analysis

Student's *t* test was used to analyze the significance in differences between 2 groups, and a *p*‐value less than 0.05 was considered statistically significant. Statistical analysis was performed using with Graphpad Prism 8. Each group was repeated in triplets, and the experiments were performed independently for three times. For quantitation of proteins in label‐free experiments, there is no consensus on the standard for screening different proteins. However, it is generally necessary to meet the double filter criteria of differential multiples and statistical analysis, such as fold change >1.5 or <0.67 and statistical significance denoted by *p*‐value <0.05.

## 3 RESULTS

### 3.1 Clinical sample collection and identification of differentially expressed proteins between IA and STA groups

Five pairs of IA/STA samples were collected for mass spectrometry and four pairs for IHC. There were no significant differences in sex, age, or risk factors between the two groups (Table S1). In proteomic analysis, a total of 1908 proteins were identified, and the expression levels of 180 proteins were significantly different between IA and STA groups. Among these 180 proteins, 88 were significantly up‐regulated, and 92 were down‐regulated in the IA group compared to the STA group. The results were shown in our previous studies.^16^, ^17^

### 3.2 Rabbit aneurysm model and identification of differentially expressed proteins between aneurysm and control groups

**FIGURE 1:**
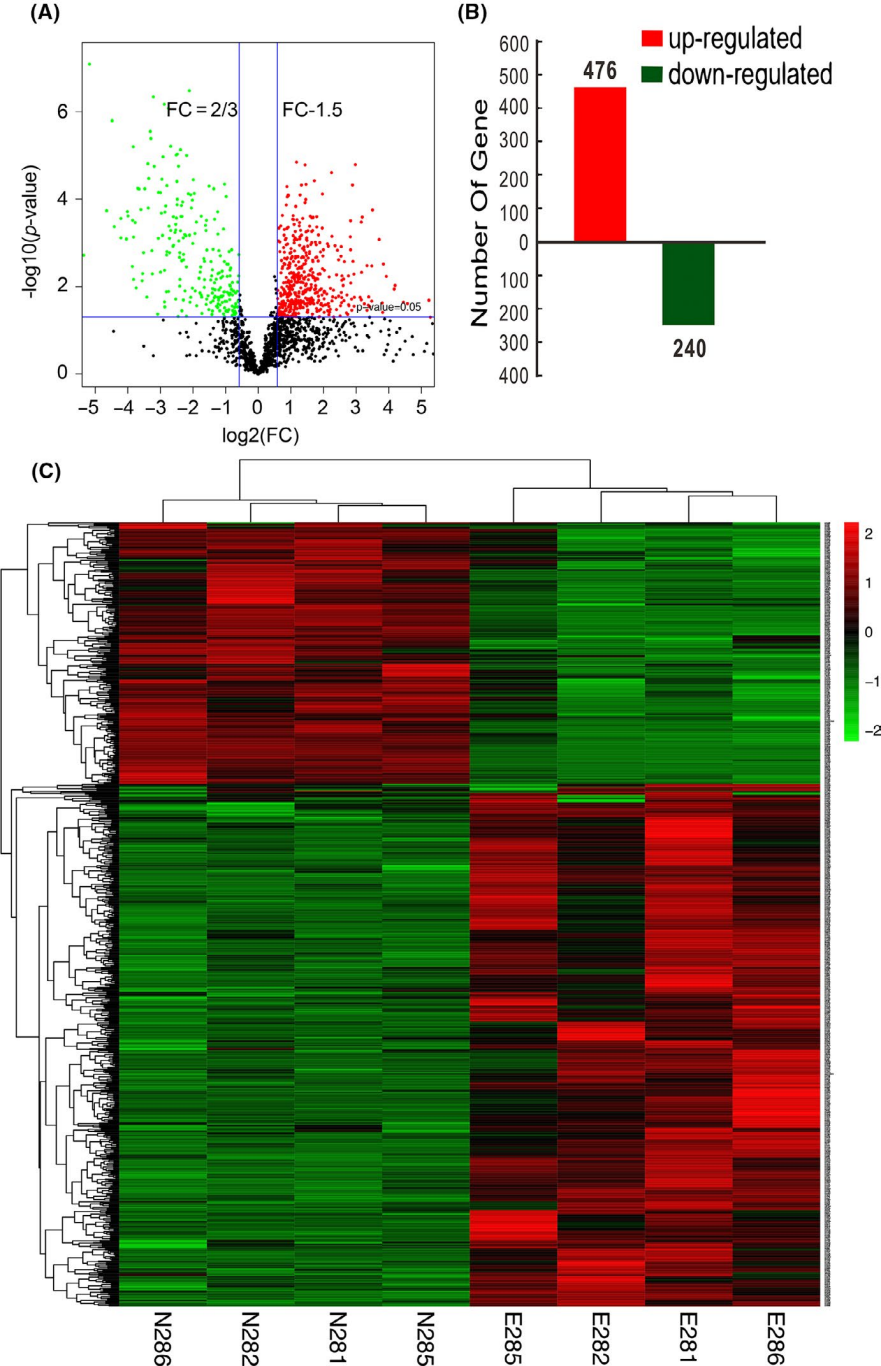
Proteome analysis of the rabbit elastase‐induced aneurysm model. (A) Volcano plot shows differential protein expression between the aneurysm group and the control group. Red dots represent up‐regulated proteins (n = 476) with *p*‐values <0.05. Down‐regulated proteins (n = 240) are labeled in green (*p* < 0.05). Black dots represent proteins whose expression levels are not significantly different. (B) Proteins with significantly different expressions between the aneurysm group and the control group. (C) Heatmap showing the comparison of the differentially expressed genes. Hierarchical clustering is shown on the left

Aneurysms were successfully induced in 10 rabbits. We identified a total of 3826 proteins from the initial set of 29822 peptides. In the significance analysis, 716 proteins were identified (Figure 1A). Out of these, 476 were up‐regulated, and 240 were down‐regulated (Figure 1B). A heatmap was presented in Figure 1C to show the comparison of the differentially expressed proteins across the 8 samples.

### 3.3 Comparison of the proteomes in rabbit aneurysm and human IA

To verify whether the rabbit aneurysm model is similar to human IA, we compared protein expression levels between these two species. A total 57 proteins were identified, of which 24 were significantly up‐regulated (Table S2), and 33 were significantly down‐regulated (Table S3). Most up‐regulated proteins were involved in immune responses and regulation of complement activation. Most of the down‐regulated proteins were involved in smooth muscle contractions.

### 3.4 Bioinformatics analysis

**FIGURE 2:**
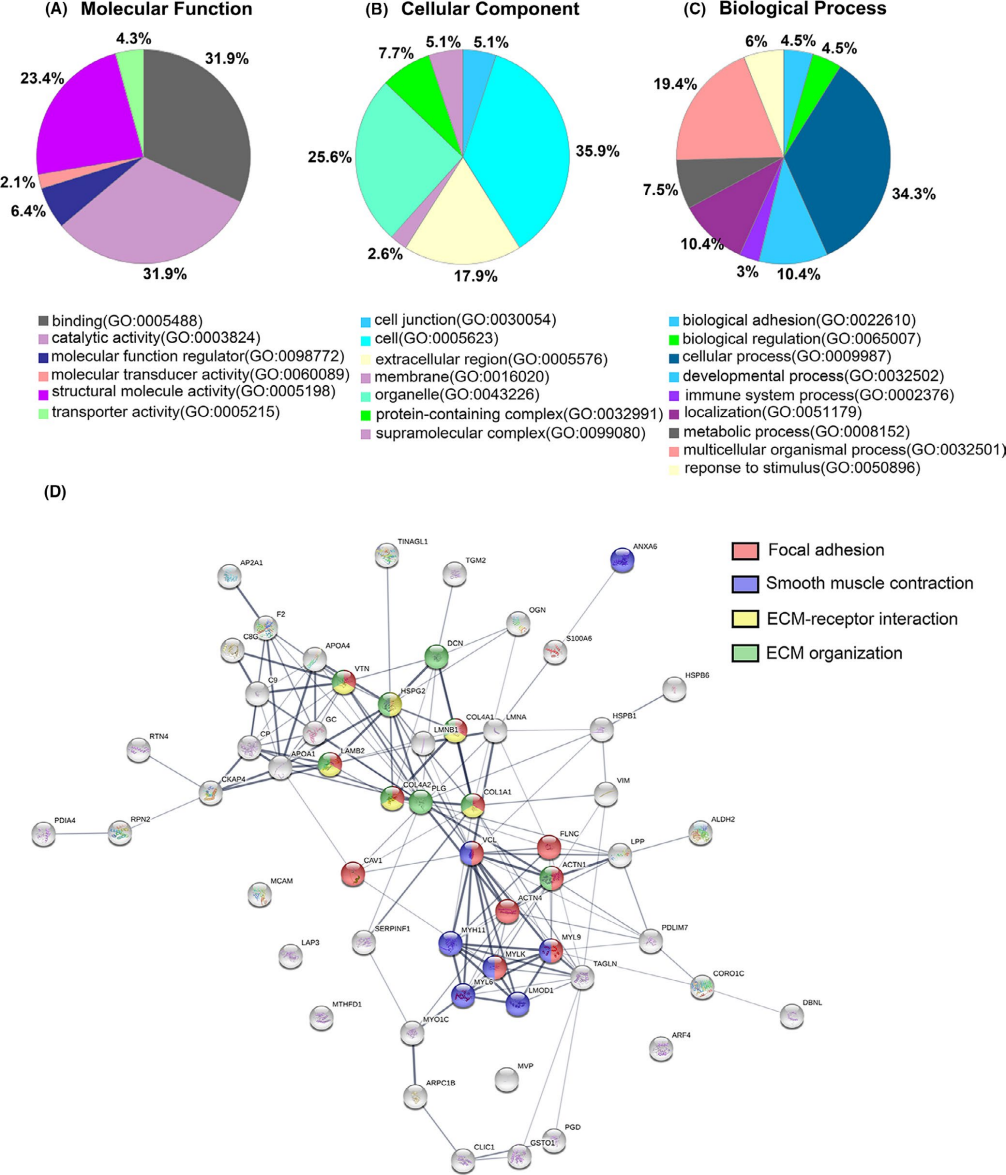
Gene Ontology (GO) classification of the 57 common proteins differentially expressed in both human and rabbit aneurysms. A, Molecular function. B, Cellular component. C, Biological process. D, Protein‐protein interaction (PPI) network by STRING. Proteins are color‐coded according to their involvement in focal adhesion pathway (red), smooth muscle contraction (blue), ECM‐receptor interaction (yellow), and ECM organization (green)

In GO analysis, 13 molecular function terms were overrepresented by the 57 proteins. Binding (GO:0005488, 31.9%), catalytic activity (GO:0003824, 31.9%), and structural molecule activity (GO:0005198, 23.4%) were found enriched in most of these genes (Figure 2A). Furthermore, most functioning parts are concentrated in cell (GO:0005623, 35.9%), organelle (GO:0043226, 25.6%), and extracellular region (GO:0005576, 17.9%) (Figure 2B). The top five most significant cellular components were in extracellular regions, such as extracellular exosome (GO:0070062), extracellular vesicle (GO:1903561), extracellular organelle (GO:0043230), extracellular space (GO:0005615), and extracellular region part (GO:0044421). Cellular process (GO:0009987, 34.3%), multicellular organismal process (GO:0032501, 19.4%), localization (GO:0051179,10.4%), and developmental process (GO:0032502,10.4%) were found enriched in most of 57 genes (Figure 2C). The top five most significant biological processes were response to wounding (GO:0009611), anatomical structure morphogenesis (GO:0009653), muscle contraction (GO:0006936), wound‐healing (GO:0042060), and muscle system process (GO:0003012). Organisms rely on proteins to implement their functions, and PPI plays a critical role in the pathogenesis of diseases (Figure 2D). We identified 12, 7, 9, and 6 proteins associated with focal adhesion, smooth muscle contraction, extracellular matrix (ECM) organization, and ECM‐receptor interaction, respectively (Table S4).

**FIGURE 3:**
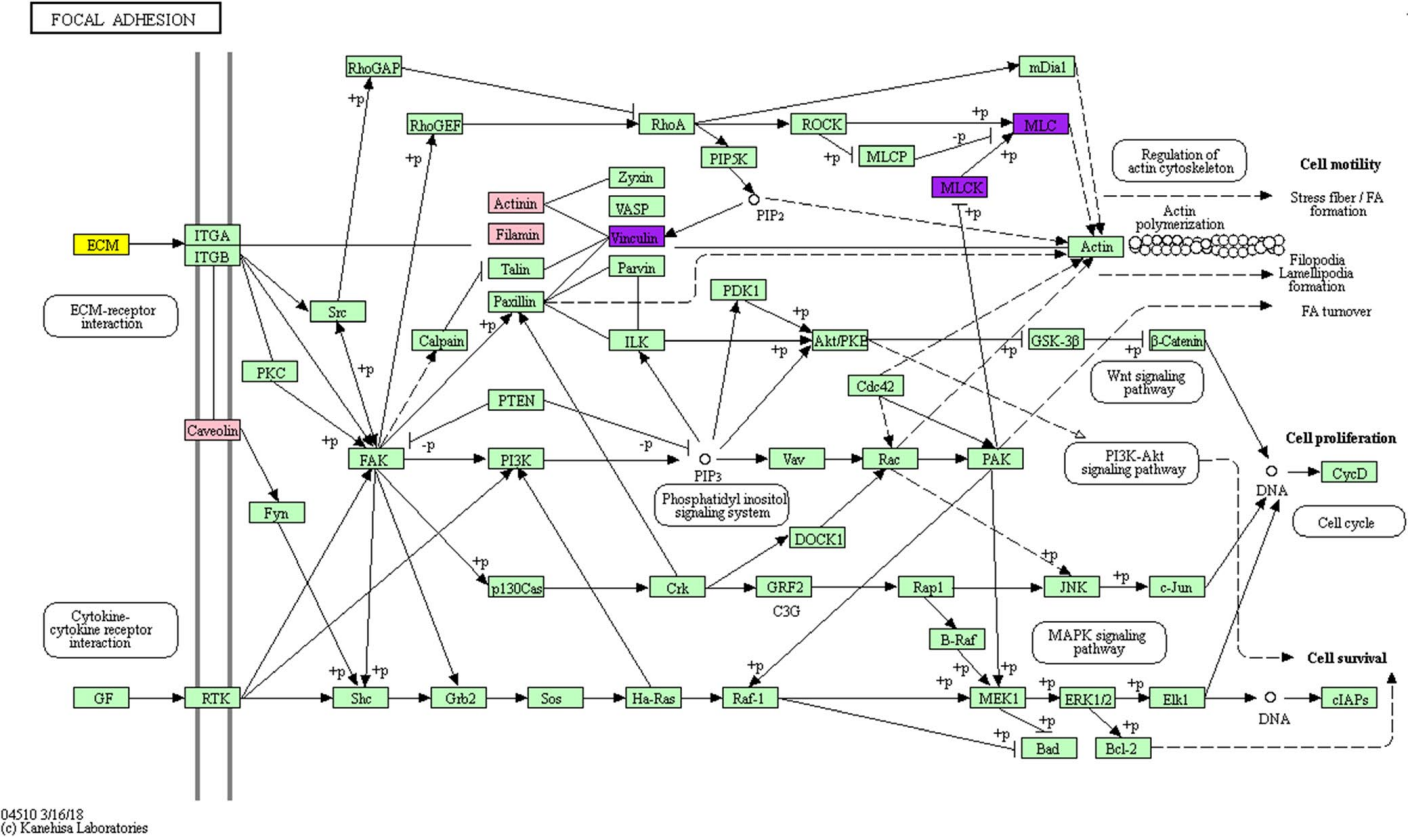
Proteins involved in the focal adhesion pathway were mapped onto KEGG pathway. Proteins that participate in both focal adhesion and ECM‐receptor interaction are highlighted in yellow. Proteins marked in purple are the ones involved in both focal adhesion and smooth muscle contraction. Proteins with significantly different expression levels in both human and rabbit aneurysms that are involved in focal adhesion are highlighted in pink

In our analysis, focal adhesion exists in both rabbit and human aneurysms. Twelve of the 57 proteins (21%) that were differentially expressed in aneurysm comparing to control in both species were related to the focal adhesion pathway. To better demonstrate its biological functions, we have highlighted the proteins related to aneurysms that were also involved in focal adhesion (Figure 3).

### 3.5 Pathological changes and validation of the aneurysm model

**FIGURE 4:**
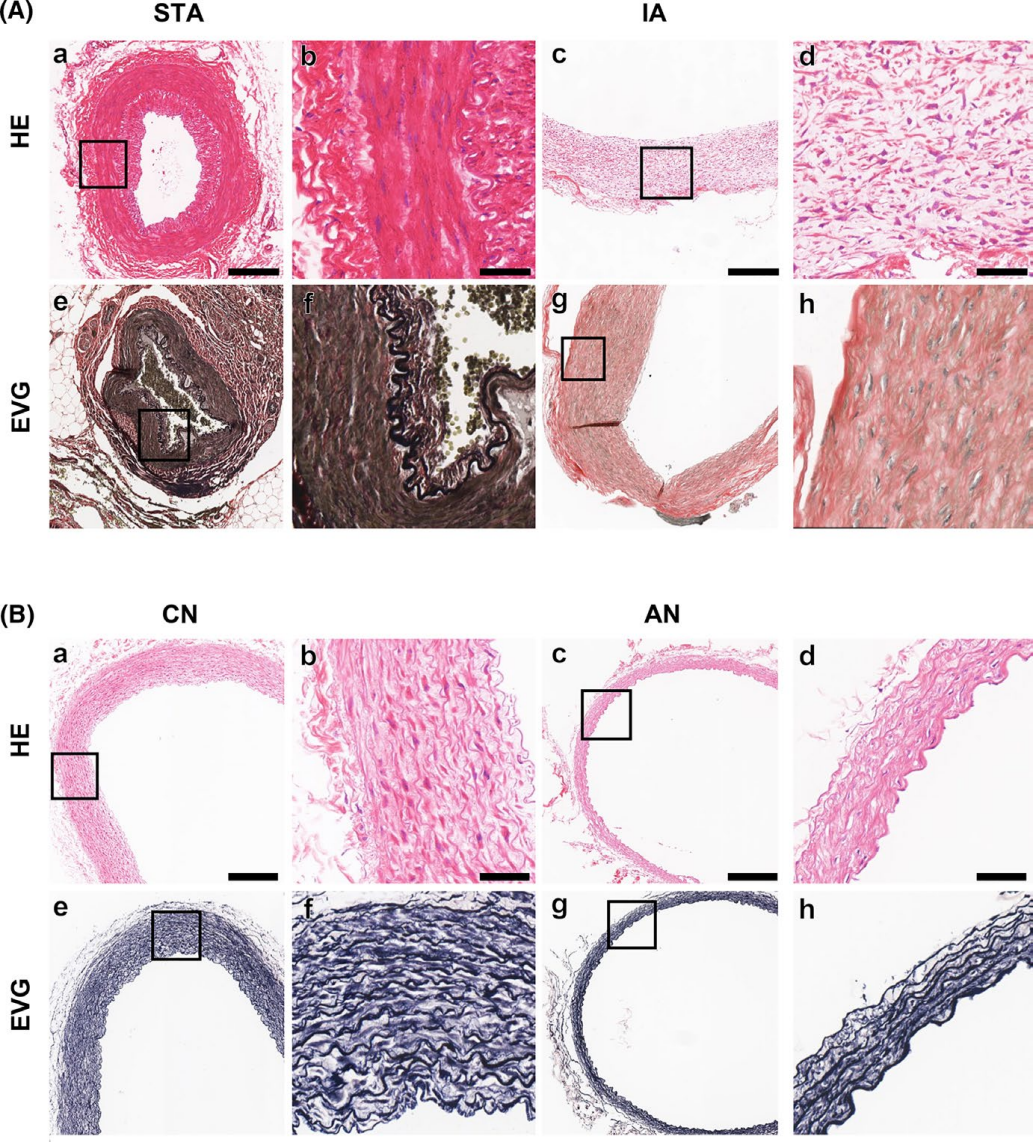
Vascular wall analysis. (A) human IA/STA vascular wall analysis: (a‐d) H&E staining on the vessel wall of STA and IA samples. (e‐h) EVG staining on the vessel wall of STA and IA samples. Bar =250 μm (a, c, e, g) or 50 μm (b, d, f, h). (B) Rabbit aneurysm and normal CCA vascular wall analysis: (a‐d) H&E staining on the vessel wall of aneurysm and control samples. (e‐h) EVG staining on the vessel wall of aneurysm and control samples. Bar =250 μm (a, c, e, g) or 50 μm (b, d, f, h)

As shown in Figure 4, HE‐ and EVG‐stained sections revealed higher integrity and a more complete and unobtrusive structure of STA (Figure 4A;a‐h). Similar results were observed in the IHC analysis on rabbit aneurysms (Figure 4B).

### 3.6 Validation of significantly differentially expressed proteins in both species identified by label‐free quantitative proteomics

**FIGURE 5:**
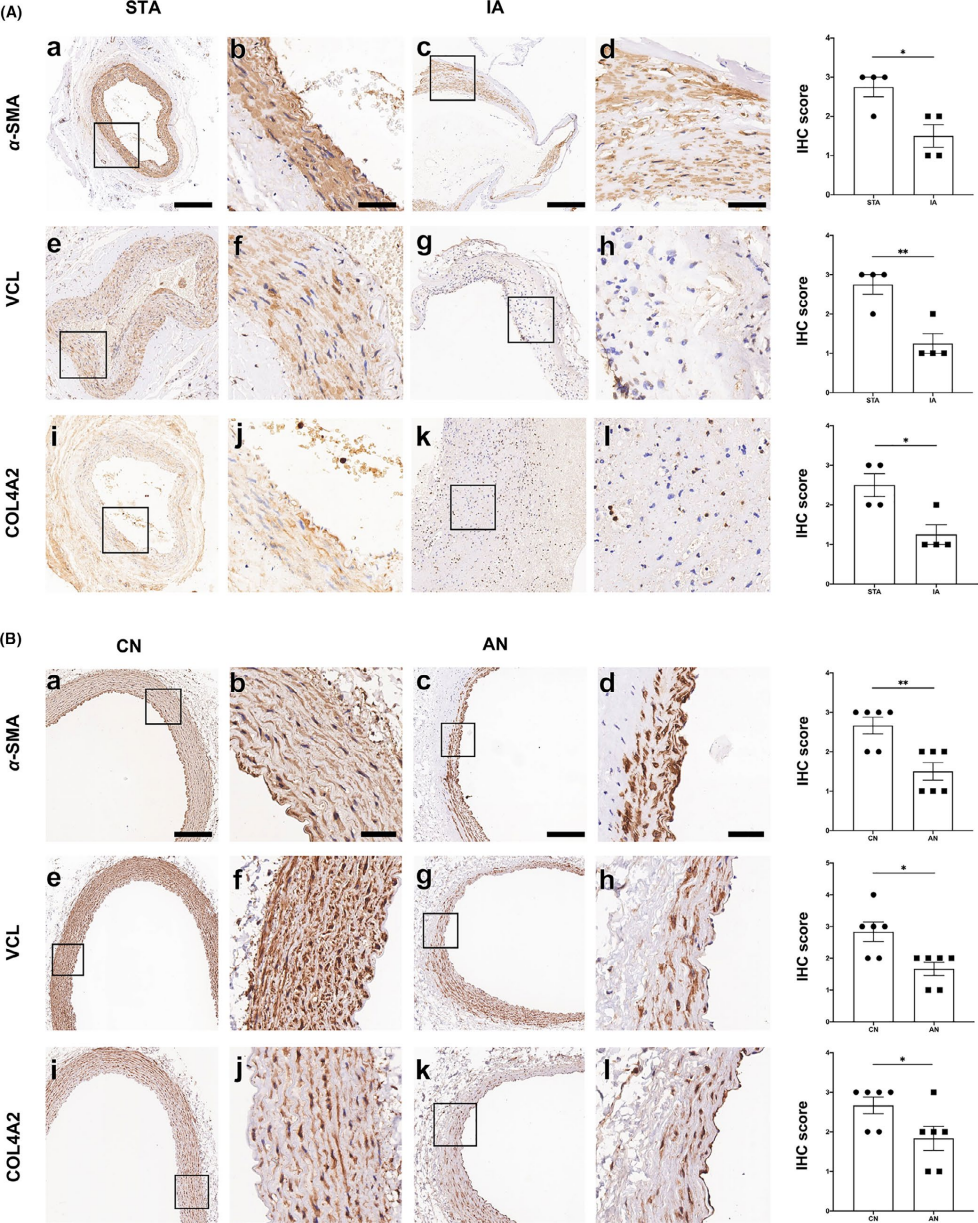
α‐SMA, VCL, and COL4A2 expression. A, α‐SMA, VCL, and COL4A2 expression in human IA/STA samples: (a‐d) α‐SMA expression in STA and IA samples. (e‐h) VCL expression in STA and IA samples. (i‐l) COL4A2 expression in STA and IA samples. Bar =250 μm (a, c, e, g, i, k) or 50 μm (b, d, f, h, j, l). (B) α‐SMA, VCL and COL4A2 expression in rabbit aneurysm and normal CCA: (a‐d) α‐SMA expression in aneurysm and control tissues. (e‐h) VCL expression in aneurysm and control groups. (i‐l) COL4A2 expression in aneurysm and control groups. Bar =250 μm (a, c, e, g, i, k) or 50 μm (b, d, f, h, j, l)

As shown in Figure 5, α‐SMA expression significantly decreased in human IA than that in STA (Figure 5A;a‐d). VCL and COL4A2 were down‐regulated in human IA compared to the STA group (Figure 5A e‐l). As shown in Figure 5B, α‐SMA, VCL, and COL4A2 were down‐regulated in AN than in the control group, similar to the human STA/IA groups.

### 3.7 Proteins related to focal adhesion and ECM pathways were down‐regulated in TNF‐α treated SMCs

**FIGURE 6:**
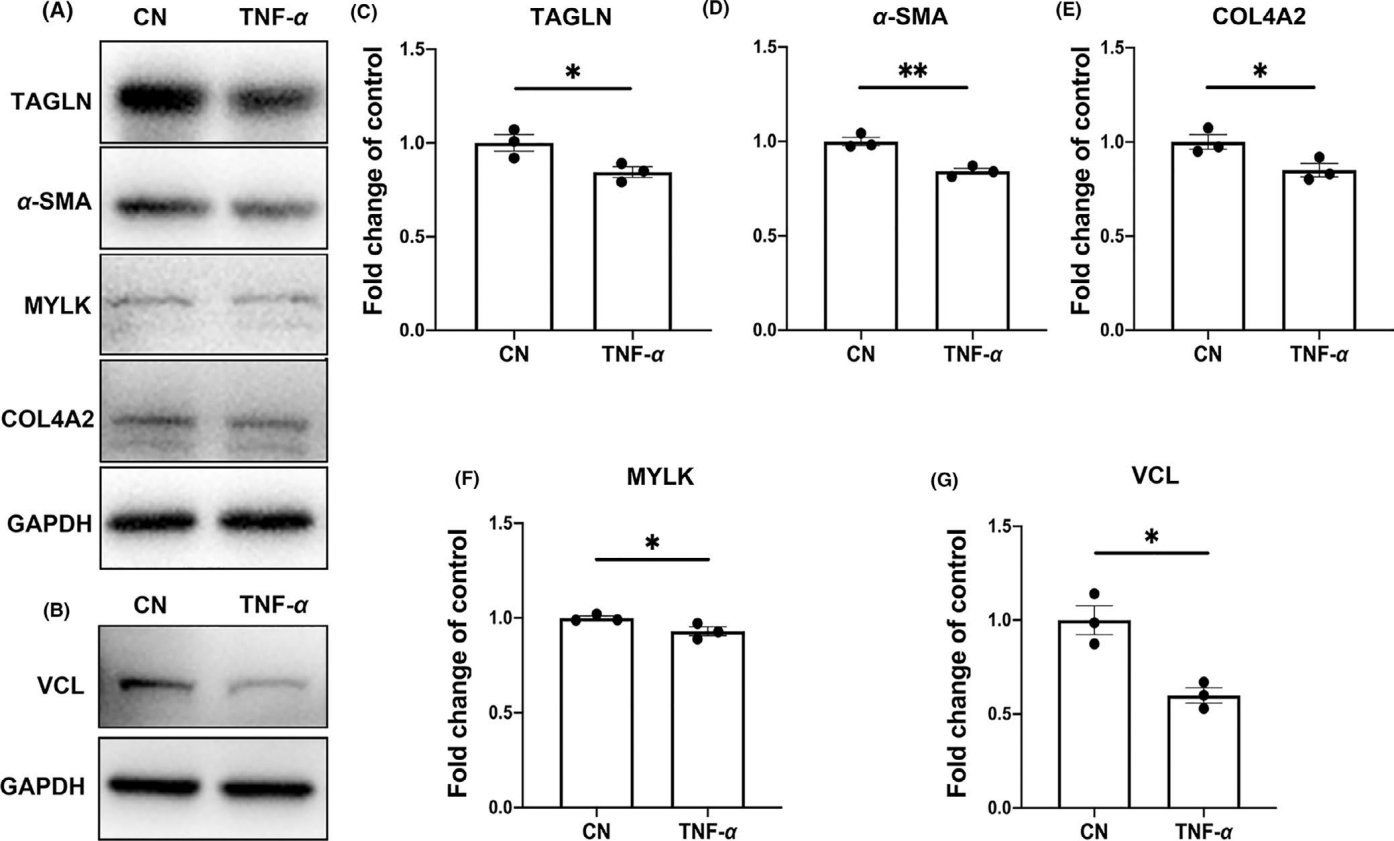
Protein expression levels in the TNF‐α‐induced SMC phenotypic modulation model. A‐G, Protein expression levels of genes of interest that were expressed differentially in both human and rabbit aneurysms, expressed as fold changes

TNF‐α was used to induce SMC phenotypic transformation (Figure S1). This model was used to evaluate the expression levels of TAGLN and α‐SMA in SMCs upon phenotypic modulation by TNF‐α (Figure 6A, C, D). After TNF‐α treatment, protein expression of contractile SMC markers TAGLN and α‐SMA was significantly decreased in SMCs (*p* < 0.05 and *p* < 0.01). The protein expression levels of COL4A2, MYLK, and VCL were decreased after SMCs were intervened with TNF‐α, consistent with the results from mass spectrometry on aneurysm samples (Figure 6A, B, E‐G).

## 4 DISCUSSION

Aneurysmal subarachnoid hemorrhage (aSAH) can cause high mortality and morbidity.^18^, ^19^, ^20^ It is believed that the wall shear stress is related to the formation of IA^21^; it can cause the damage of intracranial arteries. Oxidative stress increased after the injury of intracranial arteries,^22^ which cause a series of inflammatory reactions and further aggravate the pathological changes of intracranial artery walls. The pathological changes in IA are mostly characterized by ECM degeneration, internal elastic lamina and media disruption,^23^ and impairment of vascular SMCs.^24^, ^25^, ^26^, ^27^ The formation of aneurysms involves a series of wound‐healing processes. Once the balance between repair and damage is broken, IA is formed and may rupture eventually.^28^ In the pathogenesis of human IA, the internal elastic lamina is degenerated. In the rabbit elastase aneurysm model, the elastic lamina in the aneurysm wall is attenuated through the digestive process of elastase. This feature models the characteristics of human IA.^29^ In the elastase‐induced aneurysm model, elastase needs to be withdrawn after 20 minutes of digestion so that elastase does not enter the circulatory system. Therefore, the left CCA is theoretically unaffected and can be used as a control group. The use of the unaffected left CCA as a reliable control group has been reported in many previous studies.^13^, ^30^, ^31^ Hoh et al. ^9^ showed the histological similarity between rabbit and human aneurysms; however, such comparisons have not been performed at the protein level. Proteins exert biological functions, thus causing changes in tissues and cells. Therefore, we compared and analyzed the differentially expressed proteins in rabbit aneurysms and human IAs, hoping to find proteins that are closely linked to the pathogenesis of IA. There are multiple factors involved in aneurysm occurrence.^32^ It is generally believed that the process is related to factors including ECM degradation,^33^ disorder in SMCs, damage of intimal endothelial cells, and inflammatory cell infiltration.^26^ Among them, in addition to proteins and pathways related to ECM, proteins that constitute SMC cytoskeleton and pathways involved in the regulation of SMC phenotype during aneurysm formation is also crucial.^34^

During aneurysm formation, SMCs undergo phenotypic transformation from the contractile phenotype to synthetic SMCs. The latter possess proliferative ability but lack contractile functions. The proteins that are associated with contractility of SMCs include MYLK, TAGLN, MYH11, and collagen. MYLK is highly expressed in SMCs of the contractile phenotype, and its expression is proportional to the abundance of contractile SMCs. The absence of MYLK is known to be associated with several diseases, including aortic dissections.^35^ We previously reported that SMCs extracted from human IA showed a trend of decreased MYLK expression and decreased contractile function.^17^ SMCs depend on focal adhesion sites for their attachment to the ECM, which is crucial for effective contractions.^36^ Studies have shown that up‐regulated and down‐regulated genes are involved in the focal adhesion pathway of aneurysms.^37^ MYLK affects focal adhesion through the MYLK–MHIIA–FAK pathway or by promoting MLC phosphorylation.^38^, ^39^ Since FAK is thought to be associated with inflammatory responses,^40^ based on our previous work, we believe that FAK suppression may reduce IA formation.^41^ Collagen type IV, another essential protein present in SMCs that can stimulate the α‐SMA and SM‐MHC promoters,^42^ has been previously verified by us to be crucial in focal adhesion and ECM pathways. We found that human IA tissues had fewer SMCs and less collagen type IV.^16^ We also found that CAV‐1 is a ubiquitous gene that appears in almost every study of the rabbit aneurysm model. In our study, we not only found that CAV‐1 was down‐regulated in both rabbit and human aneurysms but demonstrated its association with the focal adhesion pathway. In addition, we have found another essential marker in the focal adhesion pathway. VCL is believed to be related to the formation of abdominal aortic aneurysm ^43^ and showed a decreasing trend in aneurysm protein spectrum in our study.

Although Laarman et al. ^44^ claimed that a better control tissue for IA gene expression studies would be cortical or circle of Willis arteries, the circle of Willis arteries themselves are prone to aneurysms. Therefore, we chose STA samples as control tissues in this study. Proteomics has been applied to explore the mechanism of many diseases.^45^, ^46^ Jiang et al. ^12^ analyzed protein expression between ruptured and unruptured IAs and found phagosomes, focal adhesion, and ECM‐receptor interaction to be the most common pathways involved in aneurysm rupture. Kleinloog et al. ^11^ used RNA sequencing to determine expression levels in 44 IA and intracranial cortical artery samples. They reported immune response pathways to be involved in ruptured aneurysms and identified lysosomes as a novel pathway in this context. Lysosomes contain a variety of hydrolases that specifically decompose various exogenous and endogenous macromolecules. The phagosome‐lysosome fusion pathway has been shown to be involved in immune responses associated with macrophages.^47^ Chyatte et al. ^48^ found macrophages, complement component 9, and T lymphocytes in the wall of the aneurysm but not in the control basilar arteries, supporting the importance of inflammatory responses and inflammatory cell infiltration in aneurysm formation. Nakaoka et al. analyzed gene expression profiles between unruptured IAs and ruptured IAs and found that most up‐regulated genes were associated with inflammatory and immune responses, whereas most down‐regulated genes were associated with mechanical of aneurysm walls.^49^ Degeneration of the aneurysm wall could be considered to be related to the lysosome pathway or to be an inflammation‐mediated effect.^11^, ^50^ Moreover, in the experiments of Holcomb et al.,^13^ 6 pairs of rabbit elastase‐induced aneurysms were used for RNA sequencing. Their results showed that the expressions of interleukin and complement system were up‐regulated, suggesting that the rabbit elastase‐induced aneurysm model could mimick inflammatory responses of aneurysm to some extent.

In the proteomic analysis of the rabbit aneurysm model, we identified differentially expressed proteins that were mainly related to focal adhesion, lysosome, and phagosome. Coincidentally, these three pathways have been shown to be related to human IA. After confirming that the MS analysis of rabbit aneurysmal proteins was consistent with that of human aneurysm proteins, we further analyzed the 57 proteins that were common to both species. We found that most of the up‐regulated proteins were involved in inflammation and complement‐related pathways. Among the 24 up‐regulated proteins, three of the top five enriched functions were related to complement and inflammation, and one was related to cytolysis.

On the other hand, down‐regulated proteins in aneurysms were mostly related to smooth muscle contraction, ECM formation, and cytoskeleton formation. Focal adhesion was identified as a pathway common to both species by pathway analysis. STRING was used for PPI analysis. Among the 57 common proteins, 12 were found to be related to the focal adhesion pathway, out of which one was up‐regulated in aneurysm and 11 were down‐regulated. All proteins involved in the smooth muscle contraction pathway were down‐regulated. These observations suggest that the phenotypic modulation of SMCs caused by aneurysms further leads to the loss of contractile functions of SMCs. Moreover, among the nine proteins involved in ECM‐related pathways, two were up‐regulated, and seven were down‐regulated. This may indicate that ECM‐related pathways participate in the wound‐healing process as well.

Elastase‐induced aneurysm in rabbits is a mature aneurysm model.^51^ Therefore, it has been used extensively in IA research for many years. In 2007, a gene chip microarray study was conducted.^52^ In the following year, Kadirvel et al ^31^ used deoxyribonucleic acid microarrays to analyze gene expression in elastase‐induced aneurysms in rabbits, along with post‐embolism aneurysm healing studies.^14^, ^53^ However, the rabbit genome has not been fully characterized, and a comprehensive genetic framework of human aneurysm is yet to be developed. Furthermore, MS‐based research on the rabbit aneurysm model has not been attempted so far. On this backdrop, we analyzed protein expression changes after inducing aneurysm in rabbits and compared them with proteins expressed in collected human aneurysm tissues, with the overall objective of arriving at a more reliable conclusion.

There are some limitations in our study. First, genes changed in rabbit aneurysms only partially overlapped with those changed in human aneurysms. We believe that this is because the genome of these species has not been fully characterized. However, among the 180 proteins identified and significantly differentially expressed in human aneurysms, there were 57 genes that were also differentially expressed in rabbit aneurysms (corresponding to a 31.5% of overlap). Secondly, the differences in gene expression in these two species should be considered as well.

## 5 CONCLUSION

In this study, we found that proteins involved in smooth muscle contraction and cytoskeletal functions were down‐regulated in aneurysms comparing to control. Markers of mature SMC phenotype decreased, suggesting that SMCs had phenotypic transformation in the aneurysms and led to changes in the vessel wall structure. We found that the expression of COL4A2, MYLK, VCL, and TAGLN decreased, all of which were involved in the focal adhesion or ECM‐receptor interaction pathways. This study cross‐compared aneurysms in two species to determine the common crucial proteins involved in both conditions. The rabbit aneurysm model induced by degrading the vascular wall with elastase has similar protein expression profiles with human aneurysms, which not only indicates the importance of structural changes in the wall of the aneurysm, but also confirms the importance of the involvement of related proteins. Our study provides basic ideas for subsequent research.

## 6 ETHICS STATEMENT

The study was reviewed and approved by the Ethics Committee of Huashan Hospital, Fudan University (2017–263). Animal experiment protocols were approved by the Department of Laboratory Animal Science at Fudan University, Shanghai, China (201802041S).

## CONFLICT OF INTEREST

The authors declare that they have no competing interests.

## AUTHOR CONTRIBUTIONS

YJL and YYS wrote the main manuscript. PLL, PXL, and KQ performed the experiments. YJL, YYS, and SCL analyzed the data, and performed bioinformatic analysis. YS, GY, and ZYF collected human tissue samples. QZA designed the model and performed DSA. WZ supervised the project and were in charge of the overall direction. All authors read and approved the final manuscript.

## Supporting information

Supplementary Material

## Data Availability

The data that support the findings of this study are available from the corresponding author upon reasonable request.
