# Supplementary material for: Comparative bioinformatics analysis between proteomes of rabbit aneurysm model and human intracranial aneurysm with label‐free quantitative proteomics
Source: CNS Neurosci Ther. 2021 Jan 3;27(1):101–12. doi: 10.1111/cns.13570 (PMC7804895; doi:10.1111/cns.13570)
Supplement: Supplementary file 1 — Supplementary Material [file CNS-27-101-s001.docx]

**Supporting Information**

**Table**

**Table S1. Characteristics of aneurysms in patients**

| No. | Gender | Age | Location of IA | Label-free | immunostaining |
| --- | --- | --- | --- | --- | --- |
| 1 | M | 43 | MCA | + | - |
| 2 | M | 50 | ICA | + | - |
| 3 | F | 69 | MCA | + | - |
| 4 | F | 45 | ACA | + | - |
| 5 | F | 38 | PICA | + | - |
| 6 | M | 78 | ACA | - | + |
| 7 | F | 61 | MCA | - | + |
| 8 | F | 49 | MCA | - | + |
| 9 | M | 59 | MCA | - | + |

ACA, anterior carotid artery; ICA, internal carotid artery; MCA, middle cerebral artery; PICA, posterior inferior cerebellar artery. F, female; M, male. Label free proteomic or immunostaining performed (+) or not performed (−).

**Characteristics of superficial temporal artery patients**

| No. | Gender | Age | Label-free | immunostaining |
| --- | --- | --- | --- | --- |
| 1 | F | 45 | + | - |
| 2 | M | 68 | + | - |
| 3 | F | 37 | + | - |
| 4 | F | 31 | + | - |
| 5 | M | 44 | + | - |
| 6 | F | 64 | - | + |
| 7 | F | 42 | - | + |
| 8 | F | 50 | - | + |
| 9 | M | 48 | - | + |

F, female; M, male. Label free proteomic or immunostaining performed (+) or not performed (−).

**Table S2. Significantly up-regulated proteins in both human and rabbit aneurysms**

| No. | Protein name | Gene name | Protein function |
| --- | --- | --- | --- |
| 1 | Lamin-B1 | LMNB1 | interleukin-12-mediated signaling pathway |
| 2 | Pigment epithelium-derived factor | SERPINF1 | endothelial cell migration |
| 3 | Protein disulfide-isomerase | PDIA4 | cell redox homeostasis |
| 4 | Major vault protein | MVP | regulation of protein tyrosine kinase activity |
| 5 | C-1-tetrahydrofolate synthase, cytoplasmic | MTHFD1 | heart development |
| 6 | Drebrin-like protein | DBNL | receptor-mediated endocytosis |
| 7 | Cytosol aminopeptidase | LAP3 | proteolysis |
| 8 | Glutathione S-transferase omega-1 | GSTO1 | regulation of cardiac muscle contraction |
| 9 | AP-2 complex subunit alpha | AP2A1 | intracellular protein transport |
| 10 | Dolichyl-diphosphooligosaccharide-protein glycosyltransferase subunit 2 | RPN2 | cellular protein modification process |
| 11 | Apolipoprotein A-IV (Predicted) | APOA4 | leukocyte cell-cell adhesion |
| 12 | Complement component C8 gamma chain | C8G | complement activation |
| 13 | Chloride intracellular channel protein | CLIC1 | regulation of cell cycle |
| 14 | Apolipoprotein A-I | APOA1 | blood vessel endothelial cell migration |
| 15 | Plasminogen | PLG | extracellular matrix disassembly |
| 16 | ADP-ribosylation factor 4 | ARF4 | cell migration |
| 17 | Prothrombin | F2 | leukocyte migration |
| 18 | Cytoskeleton associated protein 4 | CKAP4 | neutrophil degranulation |
| 19 | 6-phosphogluconate dehydrogenase, decarboxylating | PGD | oxidation-reduction process |
| 20 | Vitronectin | VTN | smooth muscle cell migration |
| 21 | Actin-related protein 2/3 complex subunit | ARPC1B | structural constituent of cytoskeleton |
| 22 | Complement component C9 | C9 | regulation of complement activation |
| 23 | Ceruloplasmin | CP | cellular protein metabolic process |
| 24 | Vitamin D-binding protein | GC | cellular protein metabolic process |

**Table S3. Significantly down-regulated proteins in both human and rabbit aneurysms**

| No. | Protein name | Gene name | Protein function |
| --- | --- | --- | --- |
| 1 | Leiomodin 1 | LMOD1 | smooth muscle contraction |
| 2 | Myosin-11 | MYH11 | smooth muscle contraction |
| 3 | Myosin light chain kinase | MYLK | smooth muscle contraction |
| 4 | Heat shock protein beta 6 | HSPB6 | regulation of angiogenesis |
| 5 | Filamin-C | FLNC | muscle fiber development |
| 6 | PDZ and LIM domain 7 | PDLIM7 | actin cytoskeleton organization |
| 7 | Lipoma-preferred partner | LPP | cell adhesion |
| 8 | Laminin subunit beta 2 | LAMB2 | cell migration |
| 9 | Myosin regulatory light polypeptide 9 | MYL9 | smooth muscle contraction |
| 10 | Caveolin-1 | CAV1 | smooth muscle contraction |
| 11 | Transgelin | TAGLN | muscle organ development |
| 12 | Protein-glutamine gamma-glutamyltransferase 2 | TGM2 | regulation of smooth muscle cell proliferation |
| 13 | Vinculin | VCL | smooth muscle contraction |
| 14 | Collagen alpha 1(IV) chain | COL4A1 | epithelial cell differentiation |
| 15 | Heparan sulfate proteoglycan 2 | HSPG2 | extracellular matrix organization |
| 16 | Heat shock protein beta 1 | HSPB1 | regulation of blood vessel endothelial cell migration |
| 17 | Myosin light polypeptide 6 | MYL6 | smooth muscle contraction |
| 18 | Decorin | DCN | extracellular matrix organization |
| 19 | Melanoma cell adhesion molecule | MCAM | cell adhesion |
| 20 | Tubulointerstitial nephritis antigen-like | TINAGL1 | cell adhesion |
| 21 | Unconventional myosin-Ic | MYO1C | positive regulation of cell migration |
| 22 | Mimecan (Osteoglycin) | OGN | regulation of smooth muscle cell proliferation |
| 23 | Collagen alpha 2(IV) chain | COL4A2 | extracellular matrix organization |
| 24 | Coronin | CORO1C | regulation of focal adhesion assembly |
| 25 | Protein S100-A6 | S100A6 | regulation of fibroblast proliferation |
| 26 | Prelamin-A/C | LMNA | cell migration |
| 27 | Alpha-actinin-1 | ACTN1 | focal adhesion assembly |
| 28 | Vimentin | VIM | collagen biosynthetic process |
| 29 | Actinin alpha 4 | ACTN4 | regulation of cell migration |
| 30 | Collagen alpha 1(I) chain | COL1A1 | blood vessel development |
| 31 | Aldehyde dehydrogenase, mitochondrial | ALDH2 | ethanol oxidation |
| 32 | Annexin | ANXA6 | muscle contraction |
| 33 | Reticulon | RTN4 | protein localization to lysosome |

**Table S4. Significant differentially expressed proteins related to focal adhesion, smooth muscle and ECM related pathways**

| Pathway | Protein name | Gene name | Ratio E/N | *p* value |
| --- | --- | --- | --- | --- |
| Focal adhesion |  |  |  |  |
|  | Myosin light chain kinase | MYLK | 0.047 | <0.001 |
|  | Filamin-C | FLNC | 0.090 | <0.001 |
|  | Laminin subunit beta 2 | LAMB2 | 0.128 | <0.001 |
|  | Myosin regulatory light polypeptide 9 | MYL9 | 0.137 | <0.001 |
|  | Caveolin-1 | CAV1 | 0.143 | <0.001 |
|  | Vinculin | VCL | 0.155 | <0.001 |
|  | Collagen alpha 1(IV) chain | COL4A1 | 0.171 | 0.001 |
|  | Collagen alpha 2(IV) chain | COL4A2 | 0.291 | 0.005 |
|  | Alpha-actinin-1 | ACTN1 | 0.422 | <0.001 |
|  | Alpha-actinin-4 | ACTN4 | 0.513 | 0.003 |
|  | Collagen alpha 1(I) chain | COL1A1 | 0.574 | 0.028 |
|  | Vitronectin | VTN | 5.801 | 0.002 |
| Smooth muscle contraction |  |  |  |  |
|  | Leiomodin 1 | LMOD1 | 0.016 | <0.001 |
|  | Myosin heavy chain 11 | MYH11 | 0.045 | <0.001 |
|  | Myosin light chain kinase | MYLK | 0.047 | <0.001 |
|  | Myosin regulatory light polypeptide 9 | MYL9 | 0.137 | <0.001 |
|  | Vinculin | VCL | 0.155 | <0.001 |
|  | Myosin light polypeptide 6 | MYL6 | 0.200 | <0.001 |
|  | Annexin A6 | ANXA6 | 0.647 | 0.008 |
| ECM organization |  |  |  |  |
|  | Laminin subunit beta 2 | LAMB2 | 0.128 | <0.001 |
|  | Collagen alpha 1(IV) chain | COL4A1 | 0.171 | 0.001 |
|  | Basement membrane-specific heparan sulfate proteoglycan core protein | HSPG2 | 0.178 | <0.001 |
|  | Decorin | DCN | 0.210 | 0.001 |
|  | Collagen alpha 2(IV) chain | COL4A2 | 0.291 | 0.005 |
|  | Alpha-actinin-1 | ACTN1 | 0.422 | <0.001 |
|  | Collagen alpha 1(I) chain | COL1A1 | 0.574 | 0.028 |
|  | Plasminogen | PLG | 4.081 | 0.004 |
|  | Vitronectin | VTN | 5.801 | 0.002 |
| ECM-receptor interaction |  |  |  |  |
|  | Laminin subunit beta 2 | LAMB2 | 0.128 | <0.001 |
|  | Collagen alpha 1(IV) chain | COL4A1 | 0.171 | 0.001 |
|  | Basement membrane-specific heparan sulfate proteoglycan core protein | HSPG2 | 0.178 | <0.001 |
|  | Collagen alpha 2(IV) chain | COL4A2 | 0.291 | 0.005 |
|  | Collagen alpha 1(I) chain | COL1A1 | 0.574 | 0.028 |
|  | Vitronectin | VTN | 5.801 | 0.002 |

**Figure**

**
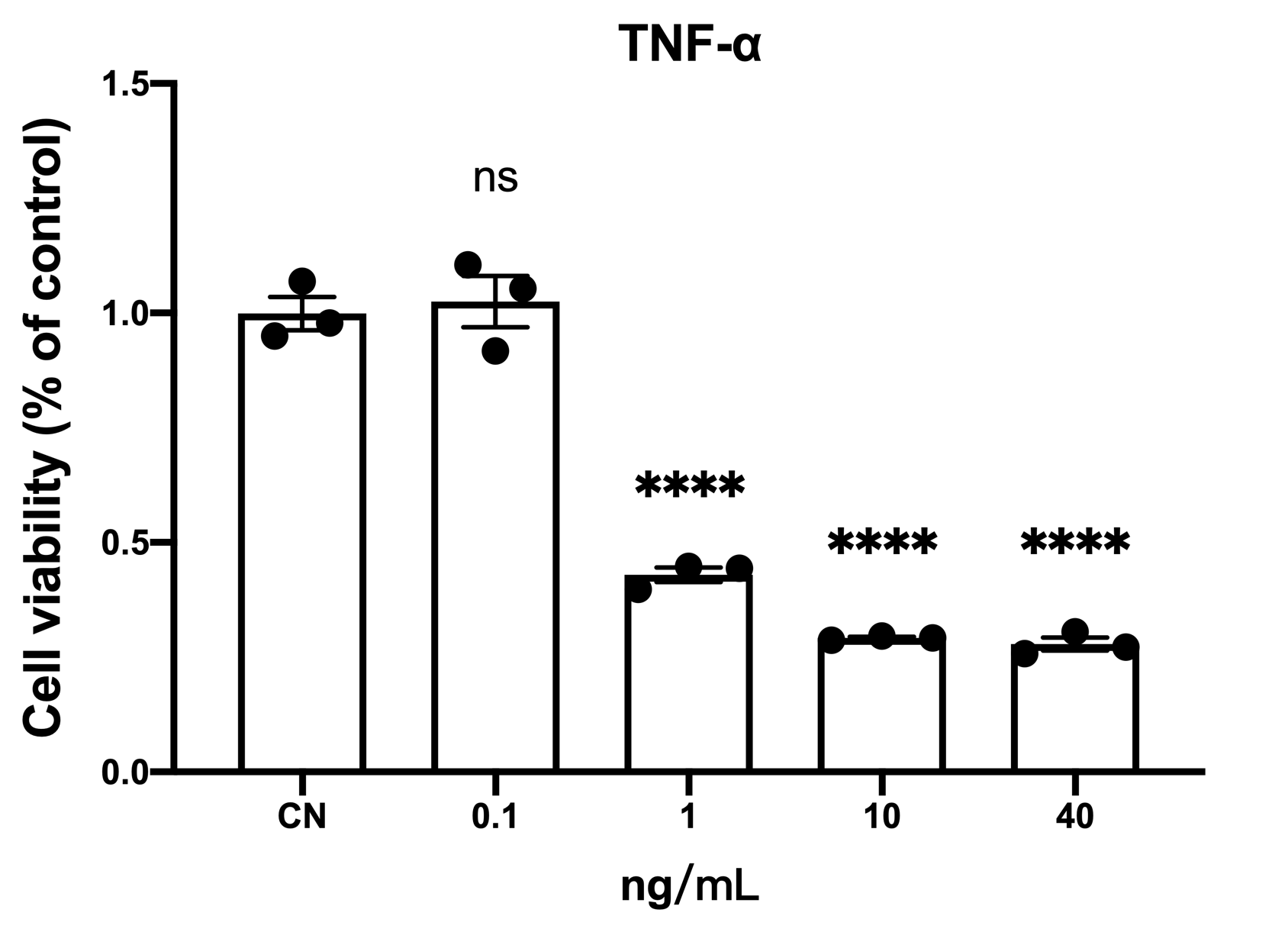
**

**FIGURE S1.** TNF-$\alpha$-induced phenotypic modulation of SMCs to model the microenvironment of aneurysm. SMC viability measured after stimulation with different concentrations of TNF-$\alpha$ for 2h decreased in a dose-dependent manner. ****, *p*＜0.0001.
